# Anniversary Discourse before the New York Academy of Medicine

**Published:** 1855-04

**Authors:** 


Art. V.—*Anniversary Discourse before the New York Academy of Medicine*, delivered in Clinton Hall, March 22d, 1854. By John H. Griscom, M. D. Published by order of the Academy.

---

The subject of this discourse is the *Relation between the People and the Science of Medicine*, a relation not as well understood as it ought to be by either of the parties. The great benefactions conferred upon the human race by medicine are not duly appreciated by the people; physicians, as a body, receive a very inadequate reward for their services. No profession gives so much of its time to objects of charity as the medical. Dr. Griscom shows that in the city of New York devoted to the cure of the sick poor, there are four general hospitals, five dispensaries, two eye and ear infirmaries, one lying-in asylum, three special hospitals, several orphan asylums and prison hospitals, besides other unenumerated charitable and penal establishments, where medical and surgical aid is rendered. In these institutions during the year 1853, there were treated 151,449 cases of disease, of every variety. Connected with these charities are 169 medical men, whose services estimated at the "lowest market value," amounted to $745,458, but were rendered, the most part, gratuitously. To speak more definitely, 36 of the number were merely boarded and lodged at the expense of the institutions, or received pay equivalent thereto, amounting in all to $6,552; 30 received salaries varying from $200 to $1500, in the aggregate $20,560; but 103 received no pecuniary compensation whatever.

"In addition to this, if we may estimate the amount of *private* gratuitous advice which every medical man renders, in the emergencies of the sick poor, at the moderate rate of $100 each per annum, the number of practitioners in this city being about 900, we have (to say nothing of bad debts) the sum of $90,000 to add to that before given, making a total of services rendered by the medical profession, in the year 1853, to the sick poor, in the city of New York of $835,458, of which there is returned $27,112."

Dr. Griscom puts the matter into the shape of an account, thus:

"January 1, 1854.

*The People of the City of New York,*

*To the Medical Profession Dr.*

To Professional Services rendered in Public Institutions, and to the poor in private, during the year 1853, $835,458

Cr. By Cash, and entertainment, 27,112

Balance, $808,346

To which we may add,

Payment not expected."

This is a pretty strong case. The people ought to look at it. It is not a solitary case. Such a state of things is not peculiar to the goodly city of Gotham; it is co-extensive with the medical profession in our country. Every where the demands upon medical men for gratuitous labor are very much greater than upon any other portion of society.

Having exhibited this fact in the strongest light. Dr. GRISCOM proceeds to speak of what the profession is attempting in the shape of sanitary measures, and prefaces his remarks with the following statistics:

"In the year 1853, there occurred in this city 22,702 deaths, of which 19,475 were from disease alone, *i. e.* exclusive of those from casualties of all kinds.

"But with a statement of the number of deaths, a mere fraction of the truth, in relation to the welfare of the people, is told. For every death, it is estimated that there are 28 cases of sickness, of an average duration of three weeks. By a comparison of the known results of sickness and death which has been made in Lancashire, England (within whose bounds are Liverpool and Manchester), in Boston, N. E., together with such data as I have been able to obtain for this city, this estimate appears to be correct, and we are thus enabled to approximate the loss sustained in this city by the pressure of disease and mortality.

"By multiplying the 22,702 deaths by 28, we have 635,656 as the number of cases of sickness which occurred during the year, and which, at three weeks duration each, gives an aggregate of time lost in 1853 by the inhabitants of New York of 36,672 years; equal to the loss of the entire lifetime of 1467 individuals, assuming the average age of our population to be 25 years, which is a liberal estimate.

"To compute the pecuniary losses incurred by this destruction of life and health, would seem an impossibility; but if we estimate the mere loss of time and the actual expenses of medicine, nursing, extra food, &c., at $1 per day, it will amount to $6,692,640."

Dr. GRISCOM is far from asserting that the principal part of all this sickness, suffering, mortality, and pecuniary loss could have been prevented by any human foresight or sagacity, but he contends with evident justice that ignorance or neglect of prophylactic measures did much to swell the fatality. He specifies intermittent fever as causing death in 29 instances, cholera infantum in 922, small pox in 681, and delirium tremens in 171. From incautious exposure to the sum 260 died, and a vast number more fell victims to diseases which might have been averted or cured.

To root out the evils connected with civil life is the problem. Such life is not necessarily provocative of disease. Men may live in crowds and yet live in the enjoyment of health. Great cities are not doomed of necessity to great physical suffering. But to ward off the thousand ills that press continually upon such communities requires energy and a knowledge of the principles of hygiene. The first step towards this, Dr. G. considers the *universal introduction of human mechanism and physiology, as a subject of study in schools.* On these subjects the people are deplorably ignorant, and until they are instructed it is impossible for them to appreciate the importance of cleanliness, temperance, and pure air. Nothing radical can be done in the way of sanitary reform until society comes to see these matters in their true light, and men learn what are the most prolific sources of disease. To accumulate information in reference to health and diffuse it among the people, Dr. Griscom suggests the establishment of a *voluntary association*, composed jointly of laymen and physicians, and recommends enforcement by law of a wise and efficient *health police.* Nothing is more certain than that law is necessary to the suppression of disease as well as of crime.

This is an able and instructive discourse and can hardly fail of results. Light must precede legislative action, and men must be awakened to their duties before they can be expected to discharge them. Dr. Griscom pours a flood of light upon all the subjects discussed in his address.

